# Exploring Posttraumatic Stress Symptoms and Posttraumatic Growth among Children Living beyond Cancer and Their Parents Using an Actor–Partner Interdependence Model

**DOI:** 10.3390/cancers14030704

**Published:** 2022-01-29

**Authors:** Amanda Wurz, Michaela Patton, Erin L. Merz, Sharon H. J. Hou, Sara Cho, Fiona Schulte

**Affiliations:** 1 School of Kinesiology, University of the Fraser Valley, Chilliwack, BC V2R 0N3, Canada; amanda.wurz@ufv.ca; 2 Department of Psychology, University of Calgary, Calgary, AB T2N 1N4, Canada; michaela.patton@ucalgary.ca; 3 Department of Psychology, California State University, Dominguez Hills, Carson, CA 90747, USA; emerz@csudh.edu; 4 Department of Oncology, Division of Psychosocial Oncology, Cumming School of Medicine, University of Calgary, Calgary, AB T2N 4N1, Canada; sharon.hou@ucalgary.ca; 5 Department of Community Health Sciences, Cumming School of Medicine, University of Calgary, Calgary, AB T2N 4Z6, Canada; sara.cho@ucalgary.ca

**Keywords:** pediatric cancer, childhood cancer survivor, survivorship, psychological health, APIM, parent-child, relationships

## Abstract

**Simple Summary:**

A diagnosis of childhood cancer, and its subsequent treatment, initiates a difficult and long-lasting experience for families which can result in posttraumatic stress symptoms. However, positive change, such as growth, may also occur. The relationship between posttraumatic stress symptoms and growth in the wake of childhood cancer is poorly understood. We sought to better understand the relationships between children’s posttraumatic stress symptoms and growth and those of their parents via a survey. The results from our study showed that the children and parents in our study were faring relatively well, reporting low levels of posttraumatic stress symptoms and moderate levels of growth. The children’s posttraumatic stress symptom score was not related to, nor did it predict their growth. The same was true for their parents wherein their posttraumatic stress symptom score was not related to, nor did it predict their growth. Notably, lower posttraumatic stress symptom scores among children were associated with greater growth in their parents, and vice versa, but the parents’ posttraumatic stress symptom score was not associated with the children’s growth.

**Abstract:**

There is a growing focus on describing both negative and positive outcomes in the wake of childhood cancer. The purpose of this study was to describe and explore the relationships between posttraumatic stress symptoms (PTSS) and posttraumatic growth (PTG) among children living beyond cancer and one of their parents. As part of a larger online survey, 113 children (M_age_ at time of study = 15.82 (*SD* = 4.81); M_age_ at diagnosis = 5.86 (*SD* = 4.66)) and one of their parents completed questionnaires assessing PTSS and PTG. Descriptive statistics were used to describe the sample and levels of PTSS and PTG. Data were *z*-transformed and analyzed using bivariate correlations and *t*-tests. An actor–partner interdependence model (APIM) was used to test whether children’s and their parents’ PTSS was associated with their own PTG (actor effect) and the others’ PTG (partner effect). PTSS was low and PTG was moderate in this sample relative to scale ranges. There were no significant differences between the children’s and their parents’ PTSS (*p* = 0.535) or PTG (*p* = 0.534). Results from the APIM showed no significant actor effects (*p* = 0.185). A significant overall partner effect (*p* = 0.020) emerged. Lower PTSS for children was associated with greater PTG for their parents (b = −0.29, *p* = 0.018), but parent’s PTSS was not associated with children’s PTG (*p* = 0.434). This sample reported similar levels of PTSS and PTG to that which has been reported in the literature. Children and their parents’ scores on PTSS and PTG measures were not significantly different from one another. Children’s PTSS was negatively associated with their parents PTG, illuminating the ways in which PTSS and PTG may be related in the context of childhood cancer. Exploring family-based strategies to reduce PTSS and enhance PTG may be warranted, though further studies are required.

## 1. Introduction

A diagnosis of childhood cancer initiates a difficult and enduring experience for families [1]. For the child diagnosed, frequent and long hospitalizations, separation from the family, and aggressive treatments can lead to adverse symptoms and side effects that range from minor discomforts to major morbidity and early mortality [2]. Consequently, many children report high levels of emotional distress and anxiety during and after treatment [3]. For parents raising children diagnosed with cancer, radical adjustments are required to their lifestyle to adapt to the demands of cancer and its treatments. Marital concerns, disruptions to family functioning, and financial burden have been reported [4], and many parents of children diagnosed with cancer experience high or heightened levels of stress, depression, and anxiety in the short- and long-term [5]. Consequently, both children affected by cancer and their parents may experience trauma-related symptomatology after treatment [6,7]. Posttraumatic stress symptoms (PTSS; [8]) can include repeated frightening thoughts, shock, fear, and feelings of helplessness. 

Nevertheless, positive change may also occur following a diagnosis of cancer. Many individuals affected by cancer associate positive life changes with their illness experience [9]. Benefit finding [10] and posttraumatic growth (PTG; [11]) are two such constructs. Benefit finding has been described as the acquisition of positive effects in response to coping with a challenging situation [10] and PTG has been defined as positive psychological changes in the wake of a crisis [11]. Both benefit finding and PTG adhere to the notion that individuals struggling with highly adverse events, such as cancer, can experience positive outcomes. Thus, for the purposes of this manuscript, benefit finding and PTG will be used interchangeably, and will be referred to as PTG. Given the tremendous impact, both negative and positive, of childhood cancer on the affected child and their parent, many researchers have explored how PTSS and PTG are related following cancer and its treatments. 

Conceptually, it may be argued that PTSS and PTG are related due to their common root in trauma [12,13]. Some researchers have reported positive [14,15,16] and negative [17] associations between PTSS and PTG amongst children diagnosed with cancer, and positive associations between PTSS and PTG among the parents of children diagnosed with cancer [18]. However, others have described no relationship between PTSS and PTG among children diagnosed with cancer [7,19], nor their parents [20]. Currently, this relatively small evidence base is fraught with mixed and null results. Efforts are needed to clarify the strength and directionality of the (possible) relationships between PTSS and PTG among children living beyond cancer and their parents. 

Further, there is a need to investigate whether children’s PTSS experience influences their parents’ feelings of PTG and vice versa. Unfortunately, exploring such relationships has been relatively rare (see [21,22,23] for notable exceptions). Researchers have typically focused on either the children’s or their parents’ experiences with PTSS or PTG separately (e.g., [7,24]), or have explored the influence of child-perceived parental factors (e.g., warmth of parenting) on children’s PTSS and PTG [19]. Such approaches may preclude a deeper understanding of the family-based nature of a childhood cancer experience, and specifically, the interactional, interdependent relationship between children and their parents. Indeed, researchers have documented that the relationship between children and their parents can affect child and parent adjustment to their own and their child’s illness (respectively; [25]) and the child’s PTSS, internalizing symptoms, and social functioning [26]. It is therefore plausible that PTSS and PTG covary in close relationships, such as between children and their parents (dyads), and that the PTSS–PTG relationship is not best understood from an individual approach. Rather, considering the mutual influences that children and parents have on one another may enhance understanding not only of one’s own PTG, but the PTG of the other person thereby advancing knowledge and offering targets for clinical practice. In other words, considering mutual influences may elucidate whether one’s PTSS impacts their own PTG (i.e., an “actor effect”) and the PTG of the other person (i.e., a “partner effect”). The actor–partner interdependence model (APIM; [27,28]) provides an analytic framework for examining such associations among members of interdependent relationships, including children and their parents. Such a model can highlight whether children’s PTSS influences their parents’ PTG, and vice versa. 

In keeping with a growing focus on describing both negative and positive outcomes in the wake of childhood cancer, there is a need to better understand the relationships between children’s and their parents’ PTSS and PTG. This study sought to explore the relationships between PTSS and PTG among children living beyond cancer and their parents using APIM, a dyadic data analysis technique that models both actor and partner effects while taking the relatedness (i.e., nonindependence) between a child and their parent into account. Specific objectives included:

(1) describing PTSS and PTG in children living beyond cancer and their parents and exploring the relationships between these variables; and,
(2) examining actor and partner effects of PTSS and PTG for children living beyond cancer and their parents.

## 2. Materials and Methods

### 2.1. Participants

Children were eligible to participate if they were: (1) diagnosed with cancer <21 years of age; (2) currently between 8 to 25 years of age; (3) at least 2 years post-treatment and at least 5 years postdiagnosis; (4) able to speak and read English fluently to complete the survey; and (5) currently residing in Canada. Exclusion criteria included: (1) having an acute medical issue such as major illness, injury, or surgery <1 year; and (2) receiving a diagnosis of psychosis or a developmental disability that would prevent independent survey completion. These criteria were selected after consideration of children’s cognitive capacity and validated age ranges for included questionnaires within the larger online survey, and the Children’s Oncology Group (http://survivorshipguidelines.org, accessed on 24 January 2022) definition of a “pediatric cancer survivor”. One parent of each of the children who met the above criteria was invited to participate. 

### 2.2. Procedures

Following Research Ethics Board approval (HREBA.CC-17-0059), children between 8 and 25 years of age, who had completed treatment for cancer, and who were living in Canada were recruited to participate in a larger, online cross-sectional study exploring well-being after treatment for childhood cancer. Potential participants were recruited during their regular, long-term follow-up care visits through the Hematology, Oncology, and Transplant Program at the Alberta Children’s Hospital and through advertisements distributed via social media and other online communications. Parents were invited to participate during clinic visits with their child, by phone, and through advertisements placed on social media websites. All potential participants completed an online prescreening eligibility form. Following this, a member of the study team contacted potential participants to describe the study further and complete additional screening to confirm eligibility. After confirming interest and eligibility, the children and one of their parents were emailed a unique link to complete an online consent form via Research Electronic Data Capture (RedCAP; [29]). After consent (and where indicated, parental consent and/or assent) was obtained, participants gained access to the secure online survey comprised of several questionnaires described in detail below.

### 2.3. Measures

#### 2.3.1. Socio-Demographic and Medical Information

Parents completed a series of closed-ended items describing their child’s current age and ethnicity, as well as their own ethnicity, marital status, and household income. In addition, medical data were gathered from children’s charts (and where unavailable, self-report) to collect information related to children’s sex, cancer diagnosis, time since treatment completion, and age at diagnosis. Finally, children’s medical characteristics (i.e., cancer diagnosis, stage, and treatment modalities) were used to categorize treatment intensity from Level 1 (*Least intensive treatments*) to Level 4 (*Most intensive treatments*), consistent with the Intensity of Treatment Rating Scale Version 3 [30]. 

#### 2.3.2. Posttraumatic Stress Symptoms (PTSS)

To assess PTSS in this sample, two measures were used: the Child Posttraumatic Stress Scale (CPSS-V; [31]) and The Posttraumatic Stress Disorder Checklist for the American Psychiatric Association’s Diagnostic and Statistical Manual of Mental Disorders (PCL-5; [32]). For children currently aged 8 to 17 years, the CPSS-V was used. The CPSS-V is a 17-item tool using a Likert scale ranging from 0 (*Not at all*) to 3 (*Five or more times a week*). Scores were summed for a total score, with higher scores indicating more severe PTSS. Internal consistency on the CPSS-V within this sample was high at 0.96. For children currently 18 to 25 years of age and all parents in this study, the PCL-5 was used. The PCL-5 is a 20-item self-report measure using a Likert scale ranging from 0 (*Not at all*) to 4 (*Extremely*). Scores were summed together to create a continuous measure of PTSS, with higher scores indicating more severe PTSS. Internal consistency for children’s and their parents’ scores on the PCL-5 were high, at 0.94 and 0.93, respectively.

#### 2.3.3. Posttraumatic Growth (PTG)

For children, the benefit finding scale from the Benefit-Burden Scale for *Children* (BBSC; [33,34]) was used. The benefit finding scale is comprised of 10 items, which use a Likert scale ranging from 1 (*Not at all true for me*) to 5 (*Very true for me*). Items were summed to provide a subscale score, with higher scores reflecting greater benefit finding. Internal consistency was high at 0.91. For parents, the Posttraumatic Growth Inventory (PTG-I; [35]), which contains 21 items, was used. Each item was rated on a Likert scale edited for this study, and that ranged from 0 (*I did not experience this change as a result of my child’s illness*) to 5 (*I experienced this change to a very great degree as a result of my child’s illness*). Total scores were calculated by summing all scores, with higher scores suggesting greater PTG. Internal consistency was high at 0.94.

### 2.4. Statistical Analyses

Data were analyzed using IBM SPSS. Of the 193 children recruited to the larger study, 113 parents agreed to participate; thus, 113 dyads were recruited and 80 were excluded from the analyses described herein due to missing data (i.e., not having parent data). Data were then screened to ensure the assumptions required for linear mixed methods were met. Normality, univariate and multivariate outliers, multicollinearity, homoscedasticity, and heteroscedasticity were explored following recommended procedures [36]. Children’s PTSS (*p* < 0.001), parent’s PTSS (*p* < 0.001), and parent’s PTG (*p* = 0.002) were not normally distributed according to Shapiro–Wilk’s test of normality. Scores were skewed on measures of children’s PTSS (negatively), parent’s PTSS (negatively), and parent’s PTG (positively). No univariate or multivariate outliers were identified. To describe the sample, descriptive statistics (frequencies, percentages, means, standard deviations) were computed for socio-demographic and medical variables. For all inferential statistics, alpha was set to 0.05.

To address the first objective, descriptive statistics were computed and bivariate correlations (Pearson’s r and Spearman’s rho) were conducted. As well, scores on measures of PTSS and PTG were *z*-transformed to standardize the metric for each construct, given the different measures used for children and parents. Paired-sample *t*-tests were then used to explore potential differences between children’s and their parent’s PTSS and PTG. To address the second objective, an APIM [37,38] for distinguishable dyads (i.e., by separate child or parent role in the family) was estimated wherein relationships between children’s PTSS and their parents’ PTG (and vice versa) were explored. This approach enabled the evaluation of processes that occur within dyads (i.e., actor and partner effects), rather than treating each member independently, as in a traditional regression model. Individual data were structured pairwise. All continuous variables used in the APIM were *z*-transformed so that values for children and their parents were based on the same metric and so that estimates would be standardized. All APIM analyses were performed via mixed linear models using residual maximum likelihood estimation to account for the hierarchical structure of the data (children and parents nested within families). Actor and partner effects were computed separately according to the distinguishing variable of their role in the dyad; children were coded as −1 and parents were coded as +1 to aid in interpretation of the estimates. Covariates of age and sex of the child were entered as between dyads variables. An initial interaction model was estimated, including the main effects of actor PTSS, partner PTSS, the distinguishing variable of role (child or parent), and the covariates to determine whether the actor and partner effects of PTSS on PTG were significant. This model also included two interaction effects of role x actor PTG and role x partner PTG to determine whether the actor and partner effects differed across children and their parents. Subsequently, a two-intercept model was estimated to obtain individual path estimates and tests of significance for each actor and partner effect within the model, though this approach did not provide the actor and partner main effects as in the interaction model. 

## 3. Results

### 3.1. Participants

Over a 19-month period, 254 eligible children were invited to participate. Of these, 61 (24%) declined, and 193 (76%) participated in the larger study. From the 193 children agreeing to participate, 113 (56%) also had a parent complete the survey. The participants in this study included the 113 children (50.44% female; 80.53% White) who had been diagnosed with leukemia, central nervous system tumors, solid tumors, or lymphomas as a child (<21 years of age) and who had a parent who agreed to participate. Children were on average 15.82 (*SD* = 4.81) years of age at the time of the study, and on average 9.32 (*SD* = 4.51) years post-treatment. Mean treatment intensity was moderate, at 2.64 (*SD* = 0.83) out of 4. Additional socio-demographic and medical information for children and their parents is presented in Table 1.

### 3.2. Describing PTSS and PTG in Children and Their Parents and Exploring the Relationship between These Variables (Objective 1)

As depicted in Table 1, children and their parents’ scores on the PTSS measures were low relative to scale ranges (Table 1), and 8 (7.62%) children and 6 (5.45%) parents met clinically significant thresholds for posttraumatic stress disorder. Scores on the measures of PTG were moderate relative to scale ranges. Table 2 shows the nonsignificant relationships between children’s PTSS and PTG (*r* = 0.05; *p* = 0.605) and parents’ PTSS and PTG (*r* = 0.14; *p* = 0.149). Exploring the relationships between children’s and their parents’ scores on measures of PTSS and PTG revealed nonsignificant correlations between children’s PTSS and their parents PTSS (*r* = 0.18; *p* = 0.065) and children’s PTG and parents’ PTG (*r* = 0.00; *p* = 0.984). Finally, there were no significant differences between the children’s and their parents scores in the measures of either PTSS (*t* = 0.62; *p* = 0.535) or PTG (*t* = 0.62; *p* = 0.534).

### 3.3. Examining the Actor and Partner Effects of PTSS on PTG for Children and Parents (Objective 2)

**Figure 1:**
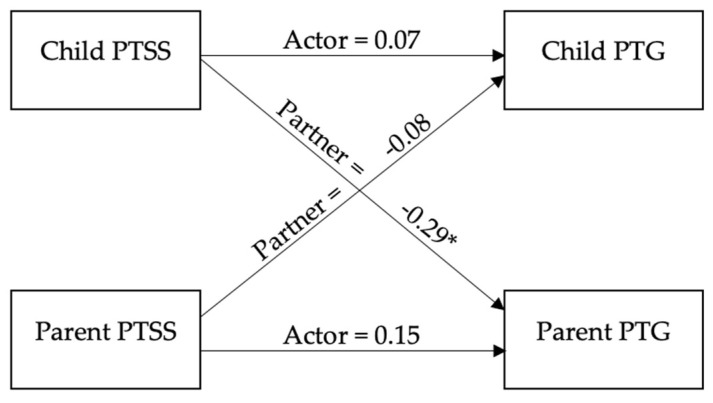
Actor−partner interdependence model results exploring posttraumatic stress symptoms predicting posttraumatic growth among child–parent dyads. Note. * *p* < 0.05.

The actor and partner effects from the APIM are depicted in Figure 1. The covariates of child sex (*b* = 0.21, *SE* = 0.14) and age (*b* = 0.00, *SE* = 0.01) were not significant (*p*s ≥ 0.153), and the main effect for role (*b* = −0.03, *p* = 0.625) was not significant, which suggests there were no mean-level differences in PTG across children and their parents. 

Actor effects were not significant (*b* = 0.11, *SE* = 0.08, *p* = 0.185), suggesting that one’s own PTSS did not predict one’s own PTG (for both children and parents). The role x actor interaction (i.e., testing whether actor effects differed based on child/parent role) was also not significant (*b* = 0.04, *SE* = 0.08, *p* = 0.643). As expected, results from the two-intercept model suggested that the individual path coefficients for children’s (*b* = 0.07, *SE* = 0.12) and parents’ (*b* = 0.15, *SE* = 0.11) actor effects were not significant (*p*s > 0.177).

Partner effects were statistically significant (*b* = −0.19, *SE* = 0.08, *p* = 0.020). However, the role x partner interaction was not significant (*b* = −0.10, *SE* = 0.08, *p* = 0.199), indicating that the partner effects did not differ based on child/parent role. However, results from the two-intercept model revealed that the path coefficient for children’s partner effect (i.e., children’s PTSS as a predictor of their parent’s PTG) was significant (*b* = −0.29, *SE* = 0.12, *p* = 0.018), but the path coefficient for parent’s partner effect (i.e., parent’s PTSS as a predictor of their child’s PTG) was not (*b* = −0.08, *SE* = 0.11, *p* = 0.434). This indicates that lower PTSS for children was associated with greater PTG in their parents, and vice versa, but that parent PTSS was not associated with children’s PTG.

## 4. Discussion

A diagnosis of childhood cancer can have a tremendous impact on the child and their parents. Understanding the negative and positive outcomes that occur in the context of childhood cancer and finding ways to enhance or optimize these aspects of mental health are therefore important for children and their parents. As a step towards better understanding the relationship between children’s and parents’ PTSS and children’s and parents’ PTG, the objectives of this study were to describe, explore, and examine the relationships between children’s and their parents’ PTSS and PTG.

Children’s scores on the PTSS measures in this study were low relative to scale ranges suggesting the children in this sample, on average, did not suffer from severe PTSS. Yet, there was a slightly higher number of children reporting any PTSS (i.e., 77%) than has been previously published in populations of individuals living beyond childhood cancer (i.e., 71%) [7]. Parents’ scores on the PTSS measure in this study, while also low, aligned with the proportions of parents of children living beyond cancer reporting clinically significant levels of PTSS in the literature [5]. Though experiencing some PTSS, the participants in this sample were seemingly faring well. In terms of PTG, scores were moderate for both children and their parents relative to scale ranges, suggesting a modest level of growth following the childhood cancer experience. These findings align with prior research describing positive outcomes (assessed via the Impact of Events and the Benefit–Burden Scales) observed in the wake of cancer [16,18]. 

Small and nonsignificant relationships were found in this study between children’s PTSS and PTG scores and parent’s PTSS and PTG scores when using bivariate correlations. This is consistent with studies published previously using similar approaches (i.e., linear regression, bivariate correlations) with children living beyond cancer [19] and children living beyond cancer and their parents [20]. A similar pattern was noted within the APIM wherein no actor effects (i.e., children’s own PTSS did not predict their own PTG scores, parents’ own PTSS did not predict their own PTG scores) were observed. It is plausible that the lack of relationship observed may be due to the individualized nature of PTSS and PTG in the context of childhood cancer, a measurement artifact, related to an extraneous variable, sample specific, and/or due to another unassessed/unexplored factor. 

An alternative interpretation of these findings is that children’s and their parents’ PTSS and PTG are indeed unrelated and the presence and magnitude of one does not predict the presence and magnitude of the other. In a study exploring PTSS and PTG among 6162 individuals who had survived childhood cancer, small relationships were also observed prompting the researchers to conclude that the relationship between the two constructs is not robust and that reconsidering the relationship between PTSS and PTG is required [7]. Thus, the findings from this study add to a mixed literature base, and highlight that concerted efforts are required to understand if and how PTSS and PTG are related. Looking ahead, studies using mixed-method approaches, wherein participants are given the opportunity to share their lived experiences regarding PTSS and PTG may be warranted. Such studies could offer a more comprehensive understanding of PTSS, PTG and related factors.

The small and nonsignificant relationships observed between children’s PTSS and their parents’ PTG (and vice versa) when using bivariate correlations is not surprising given that these analyses are less sensitive than more advanced statistical techniques, and do not take interdependence within the dyad into account. Within the APIM, children’s PTSS predicted their parents’ PTG, such that lower levels of PTSS among children living beyond cancer was associated with greater PTG in their parents. This relationship was not reciprocated from parents to children (i.e., parent’s PTSS did not predict their child’s PTG). It is worth noting that this finding emerged from the two-intercept analytic approach, but was not initially detected from the traditional interaction model, wherein the partner main effect suggested, for both children and parents, lower actor PTSS was associated with greater partner PTG. The pattern from the partner interaction effect (i.e., negative coefficient) did suggest the relationship would be more negative for children than for parents; however, the coefficient did not meet the criteria for statistical significance. While the patterns across the two approaches are concordant, the statistical difference may be related to the relatively small sample size for these analyses. As above, further efforts are still required to replicate these results and tease apart if and how children’s and parents’ PTSS is related to the others PTG. 

Nevertheless, the finding that children’s PTSS predicted their parents’ PTG when accounting for the child–parent relationship aligns with literature suggesting the relationship between children and their parents is important, and can predict both positive and negative outcomes [25,26]. This study therefore represents an important first step towards including the dyad in research on this topic. Specifically, results showed that parents’ PTSS did not predict children’s PTG, despite an observed effect of children’s PTSS on their parents’ PTG. This is a novel finding. One potential explanation for the lack of relationship between parents’ PTSS and children’s PTG may relate to the generally resilient way in which parents respond to and cope with their child’s cancer diagnosis [39] and the strategies parents use to “protect” their child from seeing their own distress [40,41], which in turn could mitigate parental influence over children’s PTG. It is also possible that children’s PTG is influenced by a number of other factors that feature more prominently in their growth. 

Clarifying the relationships among children’s and parent’s PTSS and PTG is necessary to maximize the clinical implications of this work. Findings from this study reiterate the link between children’s and parent’s mental health and reaffirm that child mental health does not occur within a vacuum. Consequently, assessing the nature of child–parent interactions in practice may be fruitful to ensuring success in therapy. Further, results suggest that strategies to mitigate or manage children’s PTSS following their cancer experience may be warranted to enhance positive outcomes among parents. As well, findings highlight that efforts are required to identify factors that predict positive outcomes, including PTG, among children living beyond cancer to inform treatment priorities and develop interventions aimed at better supporting this population. While few interventions for survivors of childhood cancer have been explored, trauma-focused cognitive behavioral therapy has been lauded for its large effect sizes among youth exposed to other traumatic events [42,43]. Thus, PTG could be considered a strength, or resilience factor, and within the context of therapy, may be a target to support shifting the narrative around traumatic memories while also enabling a deeper understanding of PTSS. 

Considering the impact of a diagnosis of childhood cancer on the child and their parents, better understanding the nature of the relationship between negative and positive outcomes is imperative. In addition to the contributions from this study, there are also important limitations to consider. First, the sample size was relatively small given the relationships being tested. Though the current sample was larger than other studies employing the APIM in pediatric health settings (e.g., [44]), it is possible potential effects were obfuscated. Given this, replication of the current findings is warranted. Second, the data were collected cross-sectionally, which precludes exploring changes or effects over time, which is important in the context of examining cancer survivorship and child–parent interactions. Researchers may consider exploring changes in PTSS and PTG over time both within-person and between-partners. Third, the sample was primarily White, amplifying an already dominant narrative. Concerted efforts are needed to capture and understand perspectives from historically excluded populations as it cannot be expected that current findings apply to all ethnic/racial groups equally. Exploring relationship and mean-level differences across children living beyond cancer and their parents with varying backgrounds is required to plan for equitable future interventions. Fourth, although the variables were informed by prior empirical work, it may be worth including additional variables, such as coping, resilience, and adjustment to better understand the range of negative and positive outcomes experienced and the effects they do/do not have within-person and between-partners. Similarly, in this study, benefit finding (acquisition of positive outcomes/benefits amidst adversity; [10]) and PTG were conflated (positive psychological changes in the wake of cancer; [11]). This decision was made on theoretical grounds, though it is possible doing so may hinder understandings of PTG. It may be worth exploring benefit finding and PTG separately in the future to explore whether these constructs are indeed interchangeable or distinct. Fifth, participants in this study were diagnosed with different cancers (blood cancer, central nervous system tumor, solid tumor, lymphoma), which could have impacted the way in which they experienced PTSS and PTG. Examining experiences with PTSS and PTG across cancer types is warranted. Lastly, it is possible that the young people recruited to this study, who were nearly 10 years since treatment, may differ those who have completed their treatment more recently. Moreover, including children, adolescents, and young adults together may have masked the different relationships each of these developmentally distinct age groups had with their own PTSS and PTG and their parents PTG. Future research could consider recruiting larger samples, across the cancer trajectory to facilitate subsample analyses wherein these relationships are explored with consideration of time since treatment and key developmental milestones for each age group (childhood, adolescence, young adulthood), which may shape parent–child interactions.

## 5. Conclusions

PTSS and PTG were low and moderate, respectively, and did not differ between children and their parents. No relationships were observed between children’s own PTSS and PTG and parents own PTSS and PTG. However, when accounting for the interactional, interdependent nature of the child–parent relationship, a partner effect was observed wherein children’s PTSS was negatively associated with their parents’ PTG. This study represents an important step towards better understanding the relationship between negative and positive outcomes and the importance of children’s influence on their parents in the wake of childhood cancer. Further research exploring PTSS and PTG is needed to inform effective supportive care options for this cohort.

## Figures and Tables

**Table 1:** Socio-demographic and medical characteristics of the sample.

Variable	Mean (*SD*) *n* (%)	Range
Child current age (years) ^†^	15.82 (4.81)	8.00–25.00
Child age at diagnosis (years) ^†^	5.86 (4.66)	0.10–17.82
Child time since treatment (years) ^†^	9.32 (4.51)	2.38–20.54
Child treatment intensity (1–4)	2.64 (0.83)	1.00–4.00
Child sex ^‡^		
Female	57 (50.44)	
Child diagnosis ^‡^		
Blood cancers	37 (32.74)	
CNS tumor	10 (8.85)	
Solid tumor	41 (36.28)	
Lymphoma	14 (12.39)	
Child ethnicity ^‡^		
White	91 (80.53)	
African Canadian	2 (1.77)	
East Asian	2 (1.77)	
Southeast Asian	1 (0.88)	
First Nations/Metis/Inuit	1 (0.88)	
South Asian	1 (0.88)	
Arab	3 (2.65)	
Latin America	1 (0.88)	
Other/mixed	9 (7.96)	
Parent ethnicity ^‡^		
White	102 (90.27)	
East Asian	2 (1.77)	
Southeast Asian	1 (0.88)	
South Asian	2 (1.77)	
Arab	2 (1.77)	
Latin American	2 (1.77)	
Household income ^‡^		
<CAD 10,000	2 (1.77)	
CAD 10,000–30,000	5 (4.42)	
CAD 30,000–50,000	7 (6.19)	
CAD 50,000–70,000	15 (13.27)	
CAD 70,000–90,000	12 (10.62)	
>CAD 90,000	70 (61.95)	
Children’s PTSS ^†^		
8–17 years	11.48 (14.81)	0.00–60.00
18–25 years	12.76 (11.68)	0.00–53.00
Parent’s PTSS ^†^	11.12 (11.03)	0.00–48.00
Children’s PTG ^†^	30.24 (10.00)	7.00–50.00
Parent’s PTG ^†^	54.82 (22.49)	5.00–97.00

Note. ^†^ mean (*SD*); ^‡^ *n* (%). CAD = Canadian dollars; *SD* = standard deviation; CNS = central nervous system; PTSS = posttraumatic stress symptoms; PTG = posttraumatic growth. Values in table represent raw scores.

**Table 2:** Relationships between study variables.

Variable	1	2	3	4	5	6	7	8	9	10
1. Child age	--	--	--	--	--	--	--	--	--	--
2. Child sex (0 = male; 1 = female) ^a^	−0.11	--	--	--	--	--	--	--	--	--
3. Child years since diagnosis	0.51 **	−0.05	--	--	--	--	--	--	--	--
4. Child time off treatment	0.56 **	−0.03	0.97 **	--	--	--	--	--	--	--
5. Child treatment intensity	0.19	0.65	0.08	0.12	--	--	--	--	--	--
6. Household income	0.14	−0.09	−0.03	0.05	0.09	--	--	--	--	--
7. Children’s PTSS	0.02	0.14	−0.12	−0.11	−0.02	0.04	--	--	--	--
8. Parent’s PTSS	−0.14	−0.03	−0.05	−0.05	−0.08	−0.07	0.18	--	--	--
9. Children’s PTG	0.06	0.17	−0.13	−0.11	0.07	−0.17	0.05	−0.06	--	--
10. Parent’s PTG	−0.12	−0.02	0.17	0.19	−0.02	−0.07	−0.18	0.14	−0.00	--

Note. ^a^ Nonparametric (Spearman’s rho); PTG = posttraumatic growth; PTSS = posttraumatic stress symptoms. * *p* < 0.05; ** *p* < 0.01.

## Data Availability

The data presented in this study are available on request from the corresponding author.
